# A new method for valuing health: directly eliciting personal utility functions

**DOI:** 10.1007/s10198-018-0993-z

**Published:** 2018-07-20

**Authors:** Nancy J. Devlin, Koonal K. Shah, Brendan J. Mulhern, Krystallia Pantiri, Ben van Hout

**Affiliations:** 1 Office of Health Economics, Southside 7th floor, 105 Victoria Street, London, SW1E 6QT UK; 2 School of Health and Related Research, University of Sheffield, 30 Regent Street, Sheffield, S1 4DA UK; 3 Centre for Health Economics Research and Evaluation, University of Technology Sydney, PO Box 123, Broadway, NSW 2007 Australia; 4 Pharmerit International, Marten Meesweg 107, 3068 AV Rotterdam, The Netherlands; 5 Pharmerit International, Enterprise House, Innovation Way, York, YO10 5NQ UK

**Keywords:** Stated preferences, Health state valuation, EQ-5D, Personal utility function, United Kingdom

## Abstract

**Background:**

Standard methods for eliciting the preference data upon which ‘value sets’ are based generally have in common an aim to ‘uncover’ people’s preferences by asking them to evaluate a subset of health states, then using their responses to infer their preferences over all dimensions and levels. An alternative approach is to ask people directly about the relative importance to them of the dimensions, levels and interactions between them. This paper describes a new stated preference approach for directly eliciting personal utility functions (PUFs), and reports a pilot study to test its feasibility for valuing the EQ-5D.

**Methods:**

A questionnaire was developed, designed to directly elicit PUFs from general public respondents via computer-assisted personal interviews, with a focus on helping respondents to reflect and deliberate on their preferences. The questionnaire was piloted in England.

**Results:**

Seventy-six interviews were conducted in December 2015. Overall, pain/discomfort and mobility were found to be the most important of the EQ-5D dimensions. The ratings for intermediate improvements in each dimension show heterogeneity, both within and between respondents. Almost a quarter of respondents indicated that no EQ-5D health states are worse than dead.

**Discussion:**

The PUF approach appears to be feasible, and has the potential to yield meaningful, well-informed preference data from respondents that can be aggregated to yield a value set for the EQ-5D. A deliberative approach to health state valuation also has the potential to complement and develop existing valuation methods. Further refinement of some elements of the approach is required.

**Electronic supplementary material:**

The online version of this article (10.1007/s10198-018-0993-z) contains supplementary material, which is available to authorized users.

## Introduction

### Background

The end product of stated preference valuation studies for patient-reported outcome (PRO) instruments is a value set (calculated via an algorithm) describing, on average for a given population, the utility decrements associated with varying levels of problems on each item (that is, each dimension, domain or attribute of health investigated) and, potentially, interaction effects between them. This generates a ‘value set’: every possible health state that can be described by the items and response options available in the PRO can be summarised by a number (to be used in the calculation of quality-adjusted life years—a generic measure of health outcome combining quality of life and length of life in a single index—these numbers should lie on a scale anchored at 0 = dead and 1 = full health), with negative values denoting states valued or modelled as worse than dead.

Standard methods for eliciting the preference data upon which these algorithms are based—discrete choice experiment (DCE; in which choices are made between two or more health states where at least one attribute is systematically varied in such a way that information related to preference parameters of an indirect utility function can be inferred), standard gamble (SG; in which living in a given health state for certain is compared to a gamble whereby the probability of living in full health is *p* and the probability of immediate death is 1 − *p*), time trade-off (TTO; in which living in a given health state for a fixed period of time is compared to living in full health for a shorter period of time) and visual analogue scale (VAS; in which health states are rated by selecting a point between the two anchor states at the ends of the scale)—vary considerably both in underlying approach and theoretical foundations. For example, while SG is grounded in expected utility theory [1], DCE arises from random utility theory [2]. TTO is often described as a more pragmatic means of proxying SG utilities, but has also been placed in the context of Hicks utility theory [3, 4]. VAS has its roots in psychology [5]. These and other established methods have been reviewed elsewhere [6, 7].

These differences in theoretical foundation have been well-described and there continues to be much debate over the relative merits of the various methods. But notably, the methods currently used to preference-weight PRO instruments (such as the EQ-5D) tend to have one important thing in common—they aim to ‘uncover’ people’s preferences by asking them evaluate a subset of health states described by the PRO, and then use their responses to infer their preferences over all dimensions and levels.

An alternative approach is to ask people to construct their own personal utility functions (PUFs). Instead of asking people to value a selection of health states, this approach involves directly asking people about the relative importance to them of the dimensions and levels described by the PRO, and potential interactions between them. In effect, the approach entails helping people to construct their own PUFs for a PRO instrument by engaging them in a series of structured tasks aimed at getting them to reflect on their preferences for different aspects of health and associated levels of severity. The aim of this paper is to describe this approach for directly eliciting PUFs, and to report the methods and findings of a pilot study to test its feasibility and acceptability for valuing a widely used generic PRO, the EQ-5D [8].

The PUF is approach has its roots in basic economics notions of utility. Specifically, the aim is to help individuals to construct their own personal utility function for health, assuming only that: (a) an economic good (in this case, health) yields utility; and (b) the more health the individual has, the greater their utility (the first two of Marshall’s axioms [9]).

The methods developed to implement the PUF approach further assume that, by a series of tasks designed to promote deliberation and reflection, the individual can meaningfully specify their utility function in a manner that reflects the marginal contribution of each argument (in this case, each EQ-5D dimension and level of severty) to their utility and the marginal rate of substitution between arguments, allowing for any possible non-linearities. The use of this information, aggregated across individual members of the general public to create a ‘social value set’, represents one means by which quality-adjusted life years (QALYs) can be estimated, consistent with the extra welfarist foundations of economic evaluation in health care [10]. We begin by explaining the rationale for developing a new approach to eliciting stated preferences. We then detail prototype methods we developed to pilot the approach, and report the results from piloting work. We conclude by highlighting the potential merits of the approach and aspects of it that require further development and testing.

### What is the matter with the current valuation approaches?

Current valuation tasks rely on survey respondents being able to imagine living in health states that they are unlikely to have ever experienced, and which are described in a highly abstract and structured way that they are unlikely to be familiar with. They have to translate the broad, generic descriptions of each health state provided into something tractable that they can think about and imagine experiencing. It is likely that this process introduces heuristics along the way—qualitative work has suggested that respondents may focus only on a subset of the dimensions presented to simplify the process [11]. Furthermore, some valuation methods then require them to reflect on what it would be like to live with those problems, unrelieved, for a certain number of years. The task is made more difficult still, because respondents often encounter what they consider to be ‘unrealistic’ health states (combinations of dimensions and levels which to them are not plausible), which affects the acceptability and realism of the task. This means that respondents cannot imagine such states, let alone value them. This whole process of ‘imagining’ health states is expected to happen within a very short time period.

In conventional stated preference valuation approaches, the purpose of the exercises is not always transparent to respondents. Interviewers typically do not reflect back the respondent’s answers to them, or check whether they agree with researchers’ interpretation of them.^1^ Engagement with the tasks is difficult to assess. The increasing popularity of DCE and online panels takes us even further in this direction, with respondents often taking a very short amount time to imagine health states and judge which they prefer.

Most fundamentally of all, current approaches rest on the assumption that respondents have a pre-existing, consistent and stable utility function over (for example) EQ-5D which we merely have to ‘tap into’ with appropriate questions. Fischoff refers to this as ‘the philosophy of articulated values’ [13]. In contrast, the ‘philosophy of basic values’ suggests that people lack clearly formulated preferences for all but the most familiar of evaluation tasks. The reality of PRO valuation studies is that respondents are constructing their utility functions on the spot, engaging in a mental production process to create responses to the tasks they are being asked to perform [14]. This is the reason that framing effects (a type of cognitive bias whereby people’s reaction to a given choice is influenced by the way is which that choice is presented [15]), and also method effects based on methodological choices relating to the tasks, are so important in stated preference studies [16]. This is clearly apparent from the extensive literature on health state valuation showing that health state values differ considerably across methods [7].

We have developed the PUF approach in an attempt to avoid some of these problems in valuing health states. The approach is designed to specifically acknowledge that respondents are constructing their preferences in response to stated preference tasks, and therefore seeks to provide opportunities for reflection and deliberation (by contrast, many valuation protocols actually prohibit respondents from changing their responses as they ‘learn’ and proceed through the valuation tasks). Hence, we are attempting to build on existing research that suggests that a more structured valuation approach in which the respondent is given time to reflect on their responses will lead to more valid responses (at the individual level) that are closer to the respondent’s ‘true’ preferences [17–21].

## Methods

### Sample and administration of survey

Initial testing was conducted with small convenience samples in England and Australia (interviews with colleagues, friends and family members; findings reported elsewhere [22]). A pre-pilot was then conducted with a larger convenience sample (*N* = 30; interviews with health outcomes professionals/colleagues of authors; findings summarised elsewhere [23]). The findings of this early pre-piloting work informed the focus of the interviewer training in the main pilot, but did not result in substantial changes to the survey or approach.

For the main pilot, data were collected from a sample of members of the UK general public. In what follows, all results are based on the UK pilot data. An Excel tool and accompanying paper booklet (described in detail below; available from the authors upon request) formed the basis for one-to-one interviews, undertaken by four interviewers working for a research agency, Accent. The interviewers completed a 1-day training course on the specifics of the methodology and procedures for the study, and were given a detailed instruction booklet (albeit not a script, as the intention was to encourage natural discussion and deliberation) to guide the interviews. The Excel tool comprised one sheet for each ‘section’ (set of tasks; see “Survey instrument”), with underlying working sheets hidden in the background. See the supplementary appendix for screenshots of the tool.

All interviews took place in the homes of respondents. The sample comprised adult members of the general public in the south of England, recruited using a ‘door-knock’ approach. Individuals were eligible for the study if they were aged 18 years or older, provided informed consent, and were deemed by the interviewers not to have a cognitive impairment that would prevent them from completing the tasks. Throughout the questions, respondents were encouraged by the interviewers to reflect on their answers and to change any previous responses if appropriate. Depending on the task, responses were recorded either in the Excel tool (by the interviewer) or the paper booklet (by the respondent), or both.

The study team followed up with the interviewers periodically during the fieldwork phase, to discuss any issues encountered and to provide further guidance. However, the data were not checked or analysed until the fieldwork had been completed.

The study was approved by the Research Ethics Committee at the School of Health and Related Research via the University of Sheffield Ethics Review Procedure.

### Survey instrument

The PUF approach combines several different techniques, drawing on previous research and existing methods such as swing weighting [24], the short form individual quality of life measure direct weighting technique (SEIQoL-DW) [25]; and the Patient Generated Index [26].

Swing weighting is a method for setting weights in a multi-attribute utility function whereby an improvement from the worst value to the best value on each criterion is described as a ‘swing’. It is frequently used in the practice of multi-criteria decision analysis [24]. The respondent identifies the most important criterion (i.e., the criterion on which they would most prefer a swing from the worse value to the best value), which is given a rating of 100. The respondent then assigns (smaller) ratings to the other criteria based on the importance of swings in those criteria relative to the swing in the most important criterion. The SEIQoL-DW is an interview-based procedure for measuring the relative importance to the respondent of nominated life areas. The respondent is asked to rate their current status in each area, and to quantify how the areas compare in importance to each other (with the total value of all weights summing to 100) using an adjustable apparatus akin to a pie chart. The Patient Generated Index is a self-administered measure that quantifies the effect of a medical condition on patients’ quality of life. The respondent is asked to identify the most important areas of their life that are affected by their condition, score each area using a 0–10 scale, and allocate points amongst the areas to reflect which are most important to determining their overall quality of life.

In this study, each respondent completed the tasks described below, in order. Note that a three-level simplification of the EQ-5D-5L [27] was used in this study. The labels of levels 1, 2 and 3 in this study corresponded to levels 1, 3 and 5 (i.e., no problems, moderate problems, extreme problems) in the EQ-5D-5L.^2^

#### Section A: warm-up tasks

Respondents were asked to self-report their EQ-5D profile (that is, they rated themselves using the EQ-5D descriptive system) and EQ-VAS rating (that is, they rated themselves using the EuroQol’s standardised VAS) twice, first for their own health on the day of the interview and then for the worst health problems they have ever experienced.

#### Section B: dimension ranking task

Respondents were asked to rank the five EQ-5D dimensions^3^ (with no reference to severity—e.g. ‘I have problems in walking about’) in order of which problems they would ‘least want to have’; ties were permitted.

#### Section C: dimension rating task

Respondents were presented with five cards, each describing an improvement (or ‘swing’) from the worst level (extreme problems) to the best level (no problems) in one of the EQ-5D dimensions. They were asked which card represented the most important or valuable improvement, assigning that improvement a rating of 100 on an accompanying 0–100 scale (where 0 represented an improvement that is not important or valuable at all). They were then asked to rate the other four improvements using the same 0–100 scale; ties (i.e., same ratings) were permitted.

**Fig. 1:**
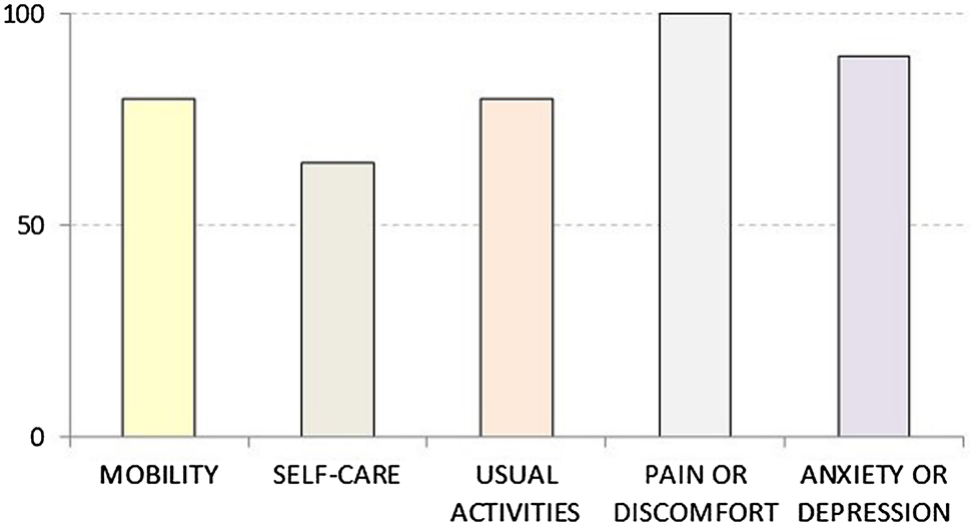
Example diagram used to represent a respondent’s section C ratings

The interviewers were encouraged to raise and discuss potential differences between respondents’ section C ratings and section B rankings. Respondents were presented with instant visual representations (bar and pie charts) of their ratings that were used to encourage reflection and comparison with their earlier responses. An example screenshot is shown in Fig. 1.

#### Section D: level rating task

For each dimension (one at a time), respondents were presented with two cards: one describing an improvement from extreme problems to moderate problems on that dimension (hereafter referred to as an ‘intermediate improvement’); the other card describing an improvement from moderate problems to no problems on that dimension. They were asked which improvement they thought was better, or if they thought that both were about the same.

The respondents were then asked to allocate 100 points in total between the two improvements, with the help of a 0–100 scale. If they considered the improvement from extreme problems to moderate problems to be better, the same as, or worse than the improvement from moderate problems to no problems, they were instructed to give the former improvement greater than 50, exactly 50, or less than 50 points, respectively. Ties (i.e., equal number of points given to intermediate improvement in multiple dimensions) were permitted.

**Fig. 2:**
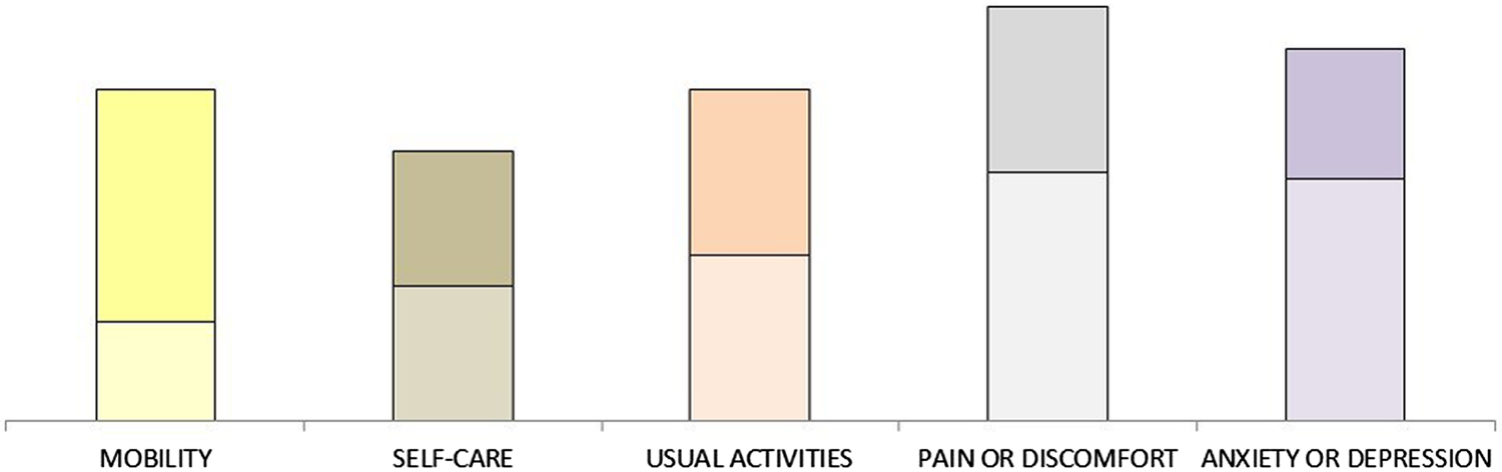
Example diagram used to represent a respondents section C and section D ratings

Respondents were presented with visual representations (weighted bar charts) of their ratings—again, these were used to encourage reflection and comparison with earlier responses. An example screenshot is shown in Fig. 2. The lighter segment of each bar represents the rating for the improvement from extreme problems to moderate problems; the darker segment represents the rating for the improvement from moderate problems to no problems.

#### Section E: paired comparison validation exercise

Respondents were presented with two paired comparison tasks, each involving a choice between two health states of unspecified duration. The tasks were generated from an algorithm based on each respondent’s previous answers, i.e., tailored to their own preferences. The algorithm started with a value of 1 (assigned to health state 11111) and applied the following decrements: for level 3 problems, a decrement equivalent to the relative weights for the relevant dimension, as determined by the respondent’s section C responses (these weights summed to 1, so the sum of decrements for 33333 reduce its value to zero); and for level 2 problems, a decrement equivalent to the relative dimension weight multiplied by the level 2 weight for that dimension, as determined by the respondent’s section D responses. This then produced an ordered ranking of health states for each respondent.

Based on each respondent’s responses to sections C and D, the first task was intended to be easier (i.e., comparing health states with a relatively large disparity in estimated personal utility) and the second task was intended to be more difficult (i.e., comparing health states which were close together in terms of estimated personal utility). A restriction was applied to the algorithm such that one health state could not logically dominate the other.

In each task, respondents were asked to choose which health state they thought was better, with no opt out or indifference option permitted—similar to the application of DCE tasks in the EuroQol protocol for the valuation of EQ-5D-5L [29].

#### Section F: search for the personal location of dead

Respondents were presented with a series of TTO-type tasks, requiring them to choose between living for 10 years in a given health state (followed by death) and living for 0 years (i.e., dying now). The health state presented in the first task was always 33333—i.e., the health state ranked 243rd (last) in terms of estimated personal utility for all respondents. Respondents choosing 33333 over immediate death were not given further choice tasks, but were asked if they could think of any health problems that were so bad that they would rather die now than live with those problems for 10 years, and if so, to describe those problems. Respondents choosing immediate death over 33333 proceeded to a second choice task in which 33333 was replaced by the health state ranked 122nd (half-way between 1st and 243rd) in terms of their personal utility function (based on their responses to sections C and D).

Five choice tasks were presented in total, with the health state presented either improved or worsened (in terms of estimated personal utility) depending on the respondent’s choice in the preceding task. Expressions of indifference were not permitted. An iterative procedure involving a bisection approach [30] was used to select the health state to be compared to immediate death. Following the fifth task, each respondent’s location of dead could be estimated to be within a range comprising 15 to 16 health states (for example, for a respondent who chose immediate death in the first task and 10 years in the health state presented in all subsequent tasks, it was deduced that they located dead between the 228th and the 243rd ranked health states).

#### Section G: examination of interactions

Respondents were presented with two paired comparison tasks, each involving a choice between two improvements in health states. In each task, both improvements described a one-level improvement in a given dimension.

Task 1 involved a choice between: (A) an improvement in the respondent’s most important dimension (as indicated in section B), with no problems in any other dimension either before or after the improvement; and (B) an improvement in the respondent’s most important dimension (as indicated in section B), with moderate problems in the respondent’s least important dimension and no problems in any other dimension either before or after the improvement. For example, a respondent whose most and least important dimensions were mobility and anxiety/depression, respectively, was presented with a choice between: (A) an improvement from 31111 to 21111; and (B) an improvement from 31112 to 21112.

Task 2 involved a choice between: (A) an improvement in the respondent’s least important dimension (as indicated in section B), with no problems in any other dimension either before or after the improvement; and (B) an improvement in the respondent’s least important dimension (as indicated in section B), with moderate problems in the respondent’s most important dimension and no problems in any other dimension either before or after the improvement. For example, a respondent whose most and least important dimensions were mobility and anxiety/depression, respectively, was presented with a choice between: (A) an improvement from 11113 to 11112; and (B) an improvement from 21113 to 21112.

Ties (expressions of indifference) were permitted in both tasks.

#### Debrief and background questions

Finally, respondents were asked a series of debrief questions, seeking feedback on the interview—in particular on aspects that respondents disliked or found difficult to understand; and background questions (gender, age and education).

### Methods of analysis

Responses to each section were analysed using descriptive methods such as means, medians, standard deviations and frequency distributions. Correlation between the rankings in section B and the implied rankings in section C was calculated using Stata’s pwcorr command. In sections D and F, preference types (identified a priori; for example, respondents who always or never gave the same ratings to intermediate improvements in section D) were assigned to respondents based on their patterns of responses.

Two methods for dealing with tied ranking data were used. The first was to take an average (AVG)—for example, if the respondent ranked MO and SC as joint number 1 and UA as number 2, this method assigns MO and SC a rank of 1.5 and UA a rank of 3. The second is to skip the next ranking in the sequence, once for each tie (EQ)—this method assigns MO and SC a rank of 1 and UA a rank of 3.

To construct the PUFs, each respondent’s personal weights over the dimensions and levels were established on a 0–1 scale. These were then anchored at dead = 0, using the section F responses. Specifically, the mid-point between the two EQ-5D states where the respondent located ‘dead’ was used, and other values were rescaled accordingly. Current methods do not allow the construction of PUFs for respondents who consider dead to lie below 33333, while for respondents who never choose A in section F, dead was assumed to lie between 11111 and the mildest health state presented to them.

The social utility function (SUF) was then reported as the mean and median of the PUFs, excluding one respondent who was deemed to be an outlier (their value of dead lay between 12221 and 11111, with a derived estimate for 33333 of − 31). No account was taken of the responses to the questions regarding possible interactions effects when deriving the SUF.

Analyses were conducted using Excel, Stata and R.

## Results

### Sample

Seventy-six interviews were conducted in December 2015. The background characteristics of the sample are summarised and compared to the general population [31, 32] in Table 1.

**Table 1:** Sample background characteristics

Characteristic	UK pilot sample	General population (%)^a^
Age (years)		
18–29	14 (18.4%)	21
30–44	28 (36.8%)	26
45–59	14 (18.4%)	25
60+	20 (26.3%)	28
Gender		
Female	49 (64.5%)	51
Male	27 (35.5%)	49
Degree or equivalent qualification		
Yes	19 (25.0%)	30
No	57 (75.0%)	70
Self-reported EQ-5D health state		
11111	46 (60.5%)	
Not 11111	30 (39.5%)	
Self-reported EQ-VAS		
Mean	79	
Median	85	

^a^Age and gender statistics taken from 2011 UK Census. Degree statistics refer to residents in England and Wales aged 16–64

Interviewers INT1, INT2, INT3 and INT4 each conducted 18, 17, 17 and 24 interviews, respectively. The sample composition varied considerably across interviewers. For example, none of the respondents interviewed by INT4 had a degree, compared to 47% of the respondents interviewed by INT3.

The interviews durations ranged from 25 to 90 min. The mean (median) duration was 46 (45) min. The mean durations by interviewer ranged from 43 to 50 min.

### Response data

#### Section A: warm-up tasks

As shown in Table 1, 60.5% of the respondents self-reported being in EQ-5D health state 11111 (no problems on any dimension). When asked about the worst health problems they have ever experienced, all respondents reported an EQ-5D profile and EQ-VAS rating worse than those describing their current self-rated health. In total, 41 states were reported by the sample when asked to describe their worst experienced health problems, spanning the dimensions and levels of the descriptive system.

#### Section B: Dimension ranking task

Ranking data are available for 75 of the 76 respondents (98.7%) and summarised in Table 2. These data were missing from the Excel tool of one respondent. Eleven respondents (14.7%) included one or more ties in their rankings. The remainder (85.3%) gave a unique rank to each of the five dimensions. All statistics suggest that, overall, pain/discomfort and mobility are the highest ranked dimensions and usual activities is the lowest ranked dimension.

**Table 2:** Summary of section B responses

	MO	SC	UA	PD	AD
Mean rank (AVG)	2.7	3.1	3.5	2.6	3.1
Mean rank (EQ)	2.6	3.0	3.5	2.6	3.0
No. times dimension was ranked top or joint top	22	11	8	26	18
No. times dimension was ranked bottom or joint bottom	10	14	24	11	18

#### Section C: dimension rating task

Rating data are available for all 76 respondents (Table 3). Nine respondents (11.8%) failed to give any dimensions a rating of 100 (recall that respondents were instructed to give a rating of 100 to the dimension they considered most important or valuable, and had the option of rating more than one dimension at 100). Two of the four interviewers had this issue in their respondents’ data. Fifteen respondents (19.7%) gave more than one dimension a rating of 100. Two of those respondents gave a rating of 100 to all five dimensions.

**Table 3:** Summary of section C responses

	MO	SC	UA	PD	AD
Mean rating	87.0	80.3	80.8	90.9	82.1
Median rating	91.0	86.5	85.0	95.0	85.0
SD rating	16.6	18.5	17.8	12.3	20.8
Implied mean rank (AVG)	2.6	3.4	3.5	2.3	3.2
Implied mean rank (EQ)	2.4	3.2	3.2	2.0	2.9
No. times dimension was given highest or joint highest rating	24	13	13	36	20

The mean and median ratings indicate that pain/discomfort and mobility are the most important dimensions. The implied rankings are similar to those provided in section B (Table 2). The correlation coefficient between mean rankings in section B and implied mean rankings in section C is 0.899 or 0.883, depending on which ranking method is used.

**Fig. 3:**
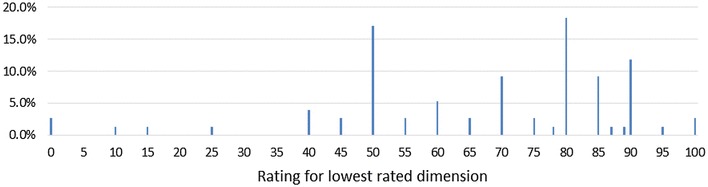
Distribution of ratings given to lowest rated dimension in section C

Most ratings given were multiples of 5, as demonstrated by Fig. 3. The mean (median) lowest rating was 67.2 (72.5). Two respondents (2.6%) gave a rating of 0 to one of the dimensions (anxiety/depression, in both cases), which implies that this dimension is completely unimportant and does not contribute to their PUF.

#### Section D: level weighting task

**Fig. 4:**
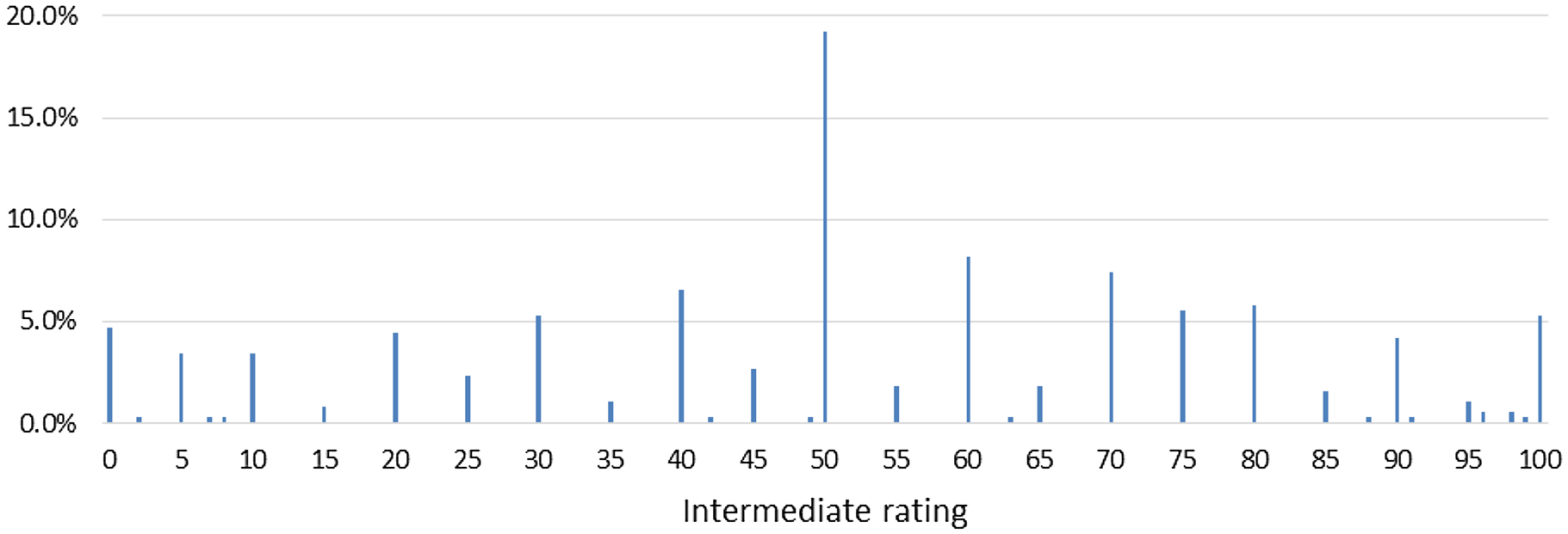
Distribution of intermediate ratings in section D (for all dimensions)

Rating data are available for all 76 respondents (Table 4). For four of the five dimensions, the median rating given to the intermediate improvement was 50. Seven respondents (9.2%) gave a rating of 50 to all five intermediate improvements. The most common approach by respondents was to give some improvements a rating of 50, some a rating of less than 50, and some a rating of greater than 50 (Table 5). A minority of respondents (10.5% in both cases) gave a rating of either 0 or 100 to at least one improvement, implying either that the improvement from level 3 to level 2 was completely unimportant (and therefore generates zero utility), or that the improvement from level 2 to level 1 is completely unimportant. Figure 4 shows the distribution of intermediate ratings, pooled across all dimensions.

**Table 4:** Summary of section D responses

	MO	SC	UA	PD	AD
Mean rating	55.2	51.3	53.3	51.1	49.7
Median rating	55.0	50.0	50.0	50.0	50.0
SD rating	28.9	25.8	26.8	29.0	27.7
No. times improvement in this dimension was given highest or joint highest rating	37	31	30	27	28

**Table 5:** Proportion of respondents following different patterns of responses in section D

	Count	%
All intermediate levels given same rating	12	15.8
All intermediate levels given different ratings	15	19.7
Mix of same and different ratings	49	64.5
All intermediate level rated at 50	7	9.2
All intermediate levels rated < 50	16	21.1
All intermediate levels rated > 50	17	22.4
Mix of ratings <, > and = 50	36	47.4
At least one intermediate level rating at 0	8	10.5
At least one intermediate level rating at 100	8	10.5

After the completion of sections A–D, interviewers were instructed to click a button in the Excel tool, designed to run a macro which prepared the tasks for sections E and F based on the respondent’s responses to the earlier sections. If the button was not clicked, the tasks for section E and F were prepared, by default, on the assumption that the respondent had given a rating of 100 to all five dimensions in section C and a rating of 50 to all five intermediate improvements in section D.

Interviewer INT2 failed to click the button in any of their 17 interviews, so the section E and F tasks presented to these 17 respondents were prepared based on the default settings rather than being tailored to their earlier responses. The other interviewers followed the instructions as intended.

#### Section E: paired comparison validation exercise

Complete choice data are available for 74 of the 76 respondents (97.3%). Data were missing from the Excel tools of two respondents.

**Fig. 5:**
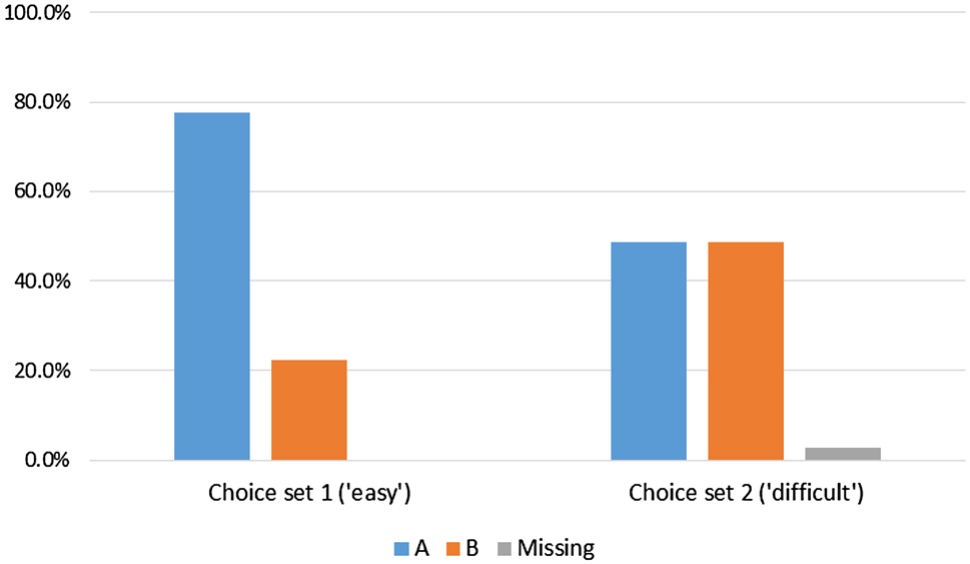
Proportions of respondents choosing A or B in the two section E tasks

In the first task, which was intended to be easier, respondents were more likely to choose A (the health state ranked higher in terms of expected personal utility) than B (the health state ranked lower in terms of expected personal utility). In the second task, which was intended to be more difficult, respondents were exactly evenly split between the two options, which were selected on the basis that they were closely ranked in terms of expected personal utility. The proportions of respondents choosing A or B in the two tasks is shown in Fig. 5.

In the majority of task 1 pairs, A had a level sum score (sum of the five dimension levels; a proxy for severity) of at least three units smaller than B—hence A could crudely be considered less severe than B. In the majority of task 2 pairs, there was no difference between the level sum scores of A and B. This demonstrates that the selection of pairs from the Excel tool algorithm worked as intended.

#### Section F: search for personal location of dead

Complete choice data are available for all 76 respondents. Table 6 summarises the responses to the section F tasks, including the number of times respondents switched between option A (i.e., preferring 10 years in the health state presented) and option B (i.e., preferring dying now/immediate death).

**Table 6:** Summary of section F responses

Choices	Number of switches	Count	%
A	Never switch	18	23.7
BBBBB	Never switch	2	2.6
BAAAA	One switch	6	7.9
BBAAA	One switch	3	3.9
BBBAA	One switch	1	1.3
BBBBA	One switch	1	1.3
BAAAB	Two switches	10	13.2
BAABB	Two switches	7	9.2
BBBAB	Two switches	3	3.9
BBABB	Two switches	3	3.9
BBAAB	Two switches	4	5.3
BABBB	Two switches	3	3.9
BABAA	Three switches	6	7.9
BABBA	Three switches	2	2.6
BAABA	Three switches	4	5.3
BBABA	Three switches	1	1.3
BABAB	Four switches	2	2.6

Eighteen respondents (23.7%) never chose B (immediate death). We can infer that for these respondents, dead lies below all of the health states defined by EQ-5D, including 33333. Fourteen of these respondents then stated they could not think of any health problems that were so bad that they would rather die now than live with them for 10 years; the remaining respondents described health states associated with locked-in syndrome, cancer and vegetative states. Two respondents (2.6%) never chose A (the health state for 10 years). We can infer that for these respondents, dead lies above the mildest health state presented to them (11113 and 12221, respectively) but we cannot determine an upper bound for the position of dead.

For the remainder of the respondents, it is possible to determine both an upper and lower bound for the position of dead within the descriptive system. For example, there were two respondents who switched choices in each of the five trade-offs (hence, their choices were ‘BABAB’). For one of these respondents, we can infer that dead lies above 32212 but below 31313. For the other, we can infer than dead lies above 31231 but below 23213.

#### Section G: examination of interactions

Complete choice data are available for 75 of the 76 respondents (98.7%). These data were missing from the Excel tool of one respondent.

The majority of respondents (72.4% in task 1; 75.0% in task 2) indicated that they thought that A was better than B (Table 7). This suggests that the value of an improvement in a given dimension depends on the levels of the other dimensions. If such ‘interactions’ were irrelevant, then we would expect more respondents to have expressed indifference between the two options. Rather, the majority of respondents indicated that a one-level improvement in a given dimension was better when no problems were present on any other dimensions than when moderate problems were present on one of the other dimensions.

**Table 7:** Summary of section G responses

	Task 1		Task 2	
	Count	%	Count	%
A	55	72.4	57	75.0
B	6	7.9	9	11.8
Indifferent	14	18.4	9	11.8
Missing	1	1.3	1	1.3

#### Feedback from respondents and interviewers

The majority of respondents provided neutral or positive responses to the debrief questions. The way in which the questions were asked was generally well-received, though one respondent expressed a preference for “straight question and answer” surveys in favour of those requiring detailed discussion. Another respondent said that they liked having the opportunity to discuss and elaborate their choices, but was not able to do so coherently for all of the questions. When probed about the reflective nature of the interviews, one respondent explained that the ranking they gave in section B differed from the ranking implied by their ratings in section C because section C referred to ‘extreme’ problems with the various dimensions whereas section B used level-free descriptors.

When asked which of the tasks were the most difficult to complete, opinion amongst respondents was split. Some respondents found the section D tasks the most difficult (e.g., because they found the task of allocating 100 points between two improvements challenging), instead preferring tasks involving simple choices between pairs of options. Others found the section E tasks the most difficult because of the difficulty in imagining the “hypothetical and unrealistic states”. Respondents who found the section G tasks the most difficult referred to the need to re-read the choice information several times, and to the fact that they could not see what the difference was between the options presented. A general theme was that respondents who preferred sections C and D rather than sections E to G felt that latter sections were difficult because there were so many factors to think about simultaneously. Opinion amongst interviewers regarding the relative difficulty of the various tasks was also split, with two interviewers identifying section D as the most difficult to explain to respondents, and one interviewer considering sections E and F to be more difficult.

Feedback was also sought on the use of diagrams, props and other materials. The diagrams (used to relay respondents’ responses to the tasks in sections C and D back to them) were generally well-received, though a few respondents noted that they did not see the point of them. Two respondents questioned the need for the 0–100 scale in section D, suggesting that the questions could be made simpler if this element was dropped. Another respondent claimed that they had initially interpreted the scale the “wrong way round” in this section. A few respondents commented that the use of physical cards in sections C and D made things difficult and overcomplicated, though a similar number of respondents claimed to have enjoyed the card-assisted tasks. Suggestions on improving the diagrams and cards (e.g., through the use of bolder colours) were received from both respondents and interviewers.

Some respondents expressed impatience about the length of the survey, while others suggested reducing the amount of repetition within and across questions.

Overall, the interviewers judged that 55 respondents (72.4%) understood and carried out the tasks easily, and that 51 respondents (67.1%) concentrated very hard and put a great deal of effort into the exercise.

### Using PUF data to estimate a social utility function

In this section, we show how the PUFs produced from our data can be used to generate an SUF (i.e., a value set). The PUF approach allows each individual’s stated preferences regarding the EQ-5D dimensions and levels, and their preferences with respect to health states worse than dead, to be quantified as a PUF anchored at 1 (full health) and 0 (dead). Using these data, a SUF is thus the aggregate of these PUFs.

As noted above, one of the interviewers consistently failed to press the button in the Excel tool which would have generated tasks E and F tailored to the respondent’s preferences generated in the previous tasks.^4^ As the responses to the tasks in F were required to anchor each respondent’s PUF to dead = 0, that interviewer’s data were dropped for the purposes of generating a value set, leaving *n* = 60 respondents.

First, responses to the tasks in sections C and D were used to generate the aggregated sample’s weights (decrements) over the dimensions and levels of the EQ-5D, on a simple 0–1 scale—as shown in Table 8.

**Table 8:** Weights for EQ-5D dimensions and levels on a 0–1 scale

	Level	Min	1st quartile	Median	Mean	3rd quartile	Max	SD	SE
Mobility	2	0.0000	0.0774	0.1092	0.1133	0.1571	0.2857	0.0630	0.0115
	3	0.0364	0.1955	0.2066	0.2061	0.2236	0.2941	0.0375	0.0069
Self-care	2	0.0000	0.0716	0.0922	0.0954	0.1200	0.2105	0.0448	0.0082
	3	0.0714	0.1745	0.1967	0.1905	0.2081	0.3125	0.0391	0.0071
Usual activities	2	0.0000	0.0736	0.0997	0.1044	0.1397	0.2857	0.0544	0.0099
	3	0.0735	0.1818	0.1929	0.1942	0.2093	0.2857	0.0359	0.0066
Pain/discomfort	2	0.0000	0.0630	0.1105	0.1104	0.1468	0.3571	0.0653	0.0119
	3	0.1266	0.1998	0.2099	0.2188	0.2346	0.3636	0.0413	0.0075
Anxiety/depression	2	0.0000	0.0568	0.0970	0.0916	0.1169	0.2353	0.0518	0.0095
	3	0.0000	0.1800	0.1939	0.1904	0.2131	0.2941	0.0526	0.0096

The mean/median level 3 decrements all sum to 1, and the decrement for a given dimension is given by calculating its relative importance, based on section C responses.^5^ The level 2 decrements are based on section D responses.^6^

**Table 9:** Social utility function (i.e., value set)

	Level	Min	1st quartile	Median	Mean	3rd quartile	Max	SD	SE
Mobility	2	0.0000	0.1238	0.1664	0.1793	0.2341	0.4706	0.1058	0.0137
	3	0.0660	0.2253	0.3025	0.3440	0.3950	0.8444	0.1639	0.0212
Self-care	2	0.0000	0.0948	0.1560	0.1600	0.2025	0.4540	0.0931	0.0120
	3	0.0714	0.2232	0.2794	0.3146	0.3391	0.7111	0.1431	0.0185
Usual activities	2	0.0000	0.1083	0.1456	0.1699	0.2251	0.4191	0.0979	0.0126
	3	0.0735	0.2203	0.2941	0.3198	0.3575	0.8000	0.1418	0.0183
Pain/discomfort	2	0.0000	0.1032	0.1600	0.1801	0.2351	0.4959	0.1197	0.0154
	3	0.1618	0.2345	0.3237	0.3653	0.4338	0.8889	0.1709	0.0221
Anxiety/depression	2	0.0000	0.0832	0.1426	0.1536	0.2145	0.3944	0.1040	0.0134
	3	0.0000	0.2091	0.2874	0.3234	0.4151	0.7556	0.1697	0.0219

The weights were then anchored at dead = 0 using the responses to section F. Of the 60 respondents, 20 indicated that 33333 (and therefore all EQ-5D health states) was not worse than dead. The remaining 40 respondents identified the position of dead within the descriptive system. Section F effectively identifies, within the individual’s utility space, the two EQ-5D states between which ‘dead’ is located. The mid-point between those two states was set at 0 and all other values were rescaled accordingly.^7^

Table 9 below reports the PUF-based value set excluding the outlier respondent (see “Methods of analysis”). The SUF derived is an average of the PUFs, and that average could be represented either by the median or mean of the PUFs [33]. Table 9 presents the SUFs for both (and, for completeness, the corresponding minimum, maximum, 1st quartile, 3rd quartile, standard deviation and standard error).

Note that the values in Tables 8 and 9 do not follow exactly from those in Tables 3 and 4. This is because Tables 3 and 4 were based on the full sample of 76 respondents, whereas Tables 8 and 9 were based on 60 respondents.

The minimum value in this SUF value set (calculated as 1 minus the utility decrement for level 3 on each dimension) is − 0.667. This compares to the minimum value of − 0.594 for the EQ-5D-3L value set for the UK (often referred to as the MVH value set) [34], and − 0.285 for the EQ-5D-5L value set for England [35]. The highest value (other than for 11111) is for state 11112, of 0.85, which is identical to the value of that state in the MVH value set. The variation in level 2 and 3 decrements across dimensions is small in the SUF value set (mean level 2 decrements range from 0.1536 to 0.1801; mean level 3 decrements range from 0.3146 to 0.3653) relative to the corresponding variations in the other value sets. The most important dimension in the SUF value set is pain/discomfort, in common with both the MVH value set and the EQ-5D-5L value set for England; followed by mobility and anxiety/depression, in common with the MVH value set. The ordering of the remaining two dimensions, self-care and usual activities, is the reverse of that in the MVH value set. Caution needs to be drawn about the implications of these differences for conclusions about the PUF approach, since our sample was small and this was intended only to be a pilot study.

## Discussion and conclusions

The PUF approach was feasible to implement, and could readily be used to generate a SUF (value set) which, even from the small sample included in this study, showed plausible characteristics. The process of deliberation and reflection appeared to work without major problems arising (according to the feedback received from respondents and interviewers), although there was evidence of interviewer effects—in part caused by the rudimentary computer-assisted tools we developed ourselves to implement the questions. Ensuring consistency across interviewers (and across studies) will be important with this method, as it is with all other stated preference approaches. Interviewer experience and training will be critical for this. The PUF approach does not eliminate (and indeed probably increases) the need for experienced, thoughtful interviewers, or for the need for quality control during data collection. The EuroQol Group has developed a set of quality control procedures to attempt to improve the quality of data collected using its protocol for valuing EQ-5D-5L health states [36]. However, since the PUF protocol is novel, we did not have many a priori expectations of what high-quality data should look like. Furthermore, the approach, by its nature, does eliminate all logical inconsistencies from the data and therefore eliminates the disordered coefficients sometimes observed in value sets based on conventional approaches [37–39].

The general PUF approach (in particular, the focus on deliberation) may have potential as a complement to (rather than a substitute for) existing approaches. It may have particular value where existing approaches to valuing PROs (e.g., as currently implemented for the EQ-5D-5L [29]) are too complicated or technology-dependent for certain populations. The PUF approach could also have applications in seeking patients’ preferences without the need to differentiate between the state they are experiencing now, and other states which are hypothetical to them, and may seem ‘unrealistic’.

In developing the study protocol, we explored a number of different approaches for the weighting tasks—ranking, numeric direct rating, VAS-type valuation, allocation of points, swing weighting—with mixed results. Some of these approaches can be described as ‘choice-based’ while others did not involve trade-offs. Still other approaches are possible, and could be improvements on the specific tasks included in our pilot study. While we opted for swing weighting for the dimension rating exercise, and allocation of points for the level weighting exercise, we do not consider there to be any need to be ‘purist’ about this: if we accept that we are helping people to construct their preferences—and acknowledge that specific methods will influence what we elicit—this may be an argument for multiple methods, constantly feeding back the results to respondents to aid their deliberation. Further research could explore whether conceptually different methods (such as those used in this study) can be combined in a coherent way, or if greater consistency in approach across tasks is desirable.

There are a number of remaining limitations to the approach reported in this paper. First, we are attempting to validate the results of our approach using the very sorts of ‘state-based’ tasks that we claim to be problematic (e.g., DCE-style pairwise choice tasks). Second, anchoring the PUF at dead still requires us to invoke a specific duration for health problems under consideration. In the study reported here, we based this on a duration of 10 years, to facilitate comparisons with existing value set protocols. Obviously, any duration could be used. But, there is no way around the need to stipulate the duration, since whether any given combination of problems is better or worse than dead may depend on its duration [40]. Third, current methods do not allow the construction of PUFs for respondents who consider dead to lie below all health states defined by the descriptive system. Fourth, the approach for obtaining information about interactions effects can be improved (as noted in “Feedback from respondents and interviewers”, these questions regarding interactions were considered difficult to understand by a number of respondents) and incorporated at an earlier stage in the process, and any data on interaction effects could be taken into account in producing a SUF value set. Fifth, the instructions provided to interviewers (e.g., to discuss potential inconsistencies with respondents) meant that interviewers may have had a strong influence on respondents’ responses, and there are limited means by which we can detect and analyse such effects. Sixth, the Excel-based tool we developed for the study could be improved considerably in functionality and presentation. Seventh, the interview is relatively long at 45 min per interview. While we obtain a lot of information per respondent, this may suggest a case for offering larger incentives and for being clear with respondents about the time commitment involved. Finally, constructing a SUF value set based on the aggregation on individual PUFs encounters some of the same conceptual challenges as the construction of social welfare functions in welfare economics: our approach here is to treat PUFs as strictly interpersonally comparable—an assumption which is of course implicit in all other stated preference methods. Furthermore, the SUF value set relies on averaging PUFs and there are a variety of ways of characterising what we mean by ‘average’ preferences [33]—the choice between which is normative.

Where next for research on the PUF approach? One direction may be to develop a more sophisticated computer-based tool with minimal need for paperwork. However, if the goal is to improve respondent engagement and to yield more considered, meaningful data, we would urge caution in the use of technology. It has been suggested that interaction elements and physical props can improve respondent engagement and understanding [41]. There is considerable scope for improving the methods used in our study, and for methodological experiments comprising direct head-to-head testing of alternative approaches. There is also scope for more sophisticated analysis of the data—e.g., in identifying and recognising preference ‘types’ in the PUFs, and reflecting those in the SUF. In the pilot study reported here, we used the PUF approach to value a simplified 3-level version of the EQ-5D-5L. The feasibility of using PUF methods to obtain values for the full EQ-5D-5L, and other more complex PRO instruments, remains to be tested.

Further research could also investigate whether the characteristics of the data observed are an artefact of the specific methods used. For example, would alternative operationalisations of the dimension rating and level rating tasks in sections C and D lead to greater variation in level 2 and level 3 decrements in the SUF? It is likely, for example, that respondents in this study were disposed to giving ratings in multiples of 10 because of ‘round number bias’ and/or the relative ease of subtracting such numbers from 100. The use of 0–100 scales can result in framing effects and there is debate around whether ratings made on such scales have interval properties [5, 42].

In addition to the potential usefulness of the overall approach, specific elements of the methods developed in this study could find applications alongside existing methods. As noted earlier, the deliberative focus of the tasks might be a useful complement to conventional state-based valuation methods. The range of states reported by respondents as their worst experienced in itself suggests the possibility of asking respondents to recall and value these states as part of ‘experience-based’ valuation approaches. The novel approach to valuing states worse than dead which we developed for this study could also find applications elsewhere, e.g., in anchoring DCE data, and may be worth exploring and further developing in its own right.

In conclusion, the use of a deliberative approach to collecting stated preference data has, we believe, some merit in generating more meaningful responses from respondents (in the sense that respondents can draw meaning from the resulting utility function and discuss/agree with, or dispute, the ways in which researchers are interpreting their preference data) and therefore reinforcing the validity and reasonableness of quality of life weights used in estimating quality-adjusted life years. This study’s contribution has been to show that such an approach appears to be feasible to use. It has the potential for use both as a standalone approach to eliciting PUFs and constructing value sets from those data, or as a complement to existing methods.

## Electronic supplementary material

Below is the link to the electronic supplementary material.

Supplementary material 1 (DOCX 935 KB)

## Footnotes

1 A ‘feedback module’—recently incorporated into the EuroQol protocol for valuing EQ-5D-5L health states [12]—is a step in this direction although limited in that it only shows how the TTO health states have been ranked, not how the values themselves are interpreted and used to generate a utility function.

2 The rationale for this was that we considered the wording of the EQ-5D-5L labels to be more appropriate than those of the EQ-5D-3L labels (e.g. the 5L label for the worst mobility level—‘unable to walk about’ appears to be an improvement over the corresponding 3L label—‘confined to bed’ [27]), and that our ultimate ambition is to apply the method to generate utility functions for the EQ-5D-5L.

3 Mobility; self-care; usual activities; pain/discomfort; anxiety/depression.

4 This was a limitation with the (relatively rudimentary) Excel tool we developed for this study. If the PUF approach was to be taken forward, it would be a simple matter to automate this step, so that it is not subject to interviewer oversight.

5 For example, if mobility had a mean rating that was 25% of the sum of all five mean ratings, then MO level 3 would be given a mean decrement of 0.25 in Table 9.

6 For example, if the mean level 2 rating for mobility was 50, and the mobility level 3 decrement was 0.25, then the mobility level 2 decrement would be 0.25 * 0.5 = 0.125.

7 For example, if a respondent’s location of dead was found to lie between two health states which had 0–1 scale values of 0.45 and 0.55, then we would infer that their approximate location of dead is at 0.5. Since dead needs to be 0, all the decrements would be re-scaled accordingly. In the simple example of dead being re-scaled from 0.5 to 0, all of the decrements would double in size. Once this has been done for each respondent, Table 9 can be produced in a similar manner to Table 8.
