# Supplementary figures and images for: A new method for valuing health: directly eliciting personal utility functions
Source: Eur J Health Econ. 2018 Jul 20;20(2):257–70. doi: 10.1007/s10198-018-0993-z (PMC6438932; doi:10.1007/s10198-018-0993-z)

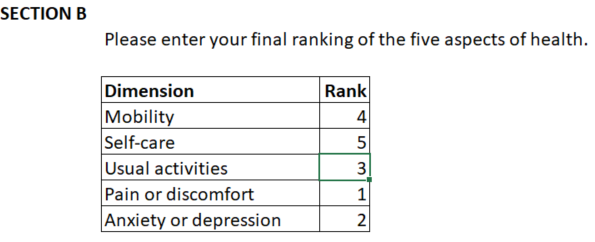


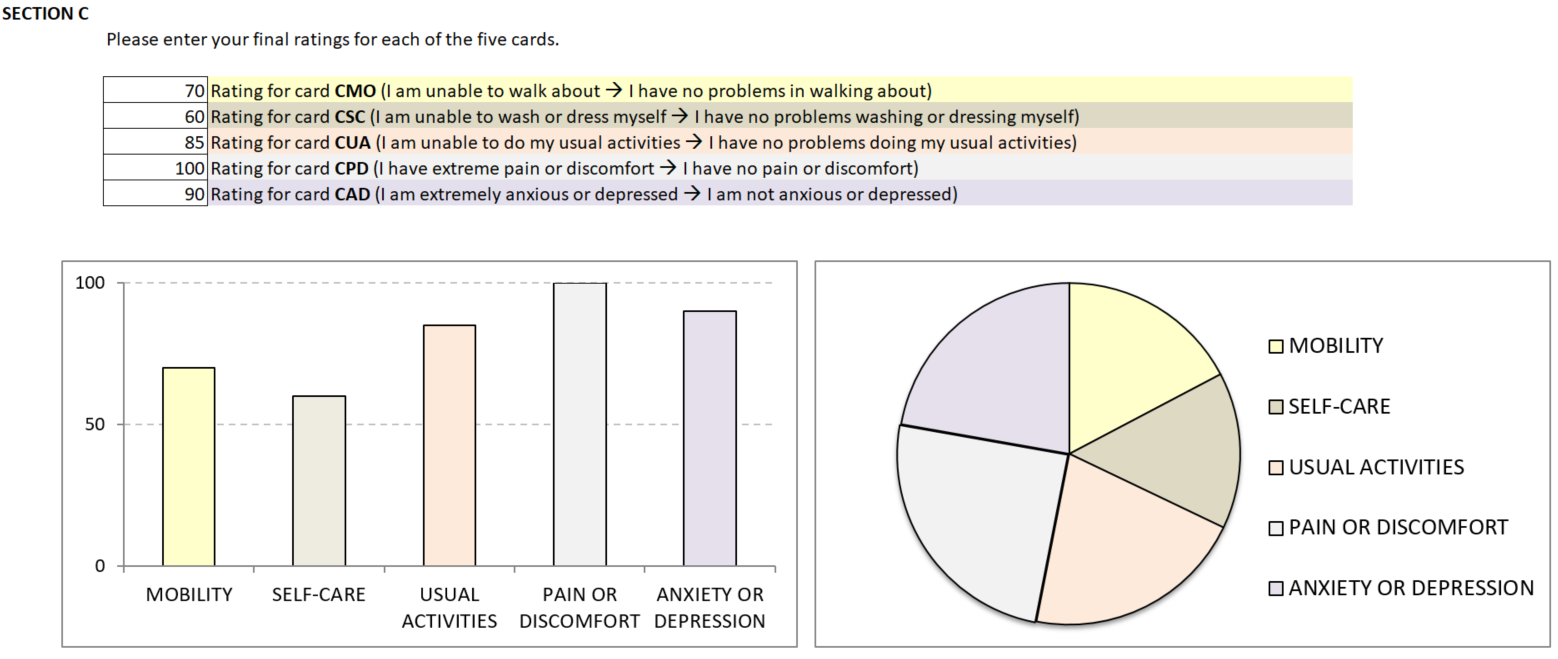


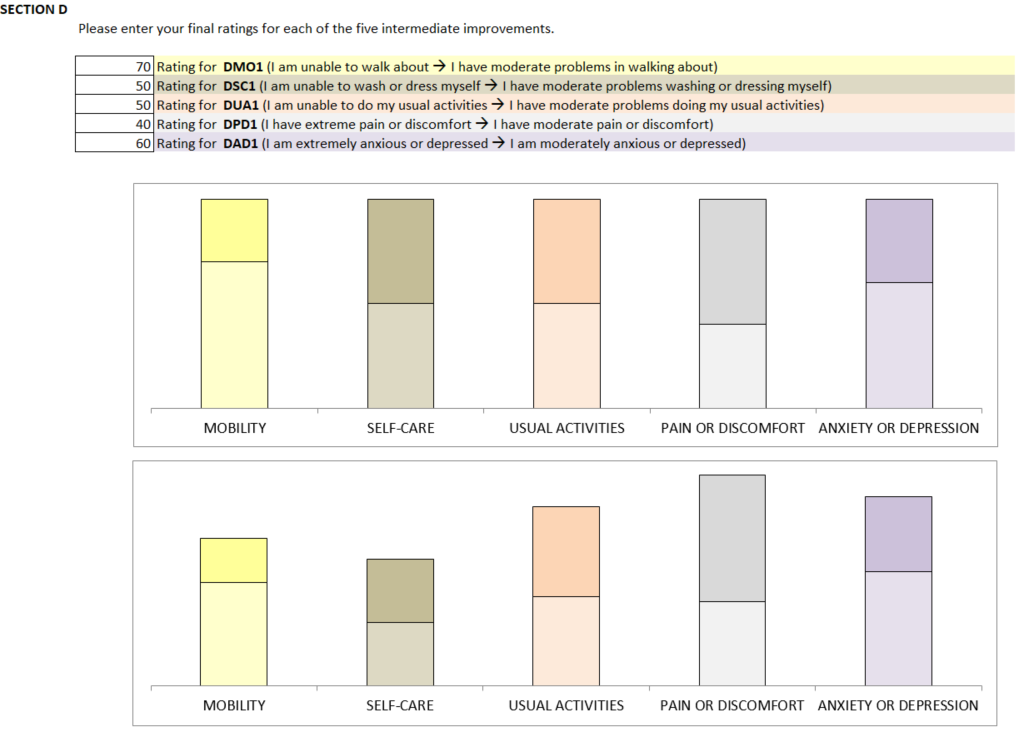


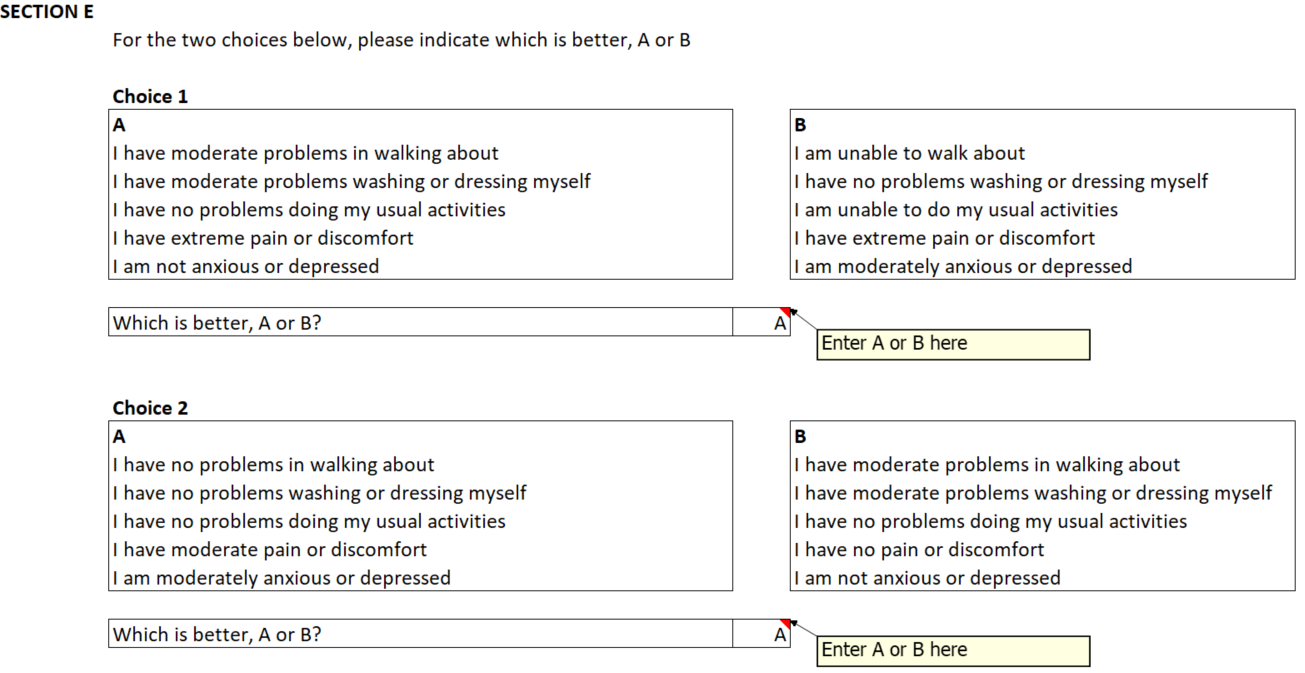


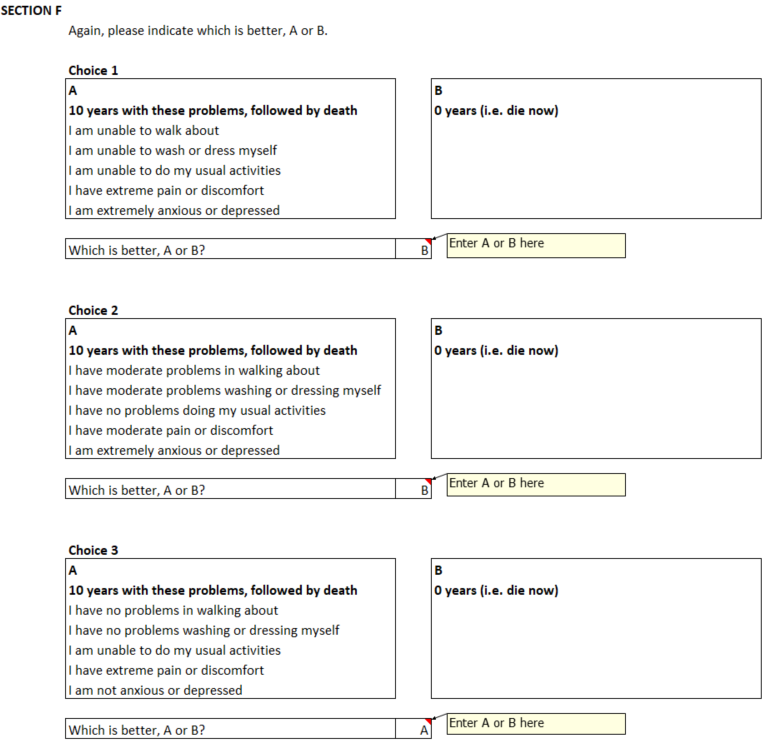


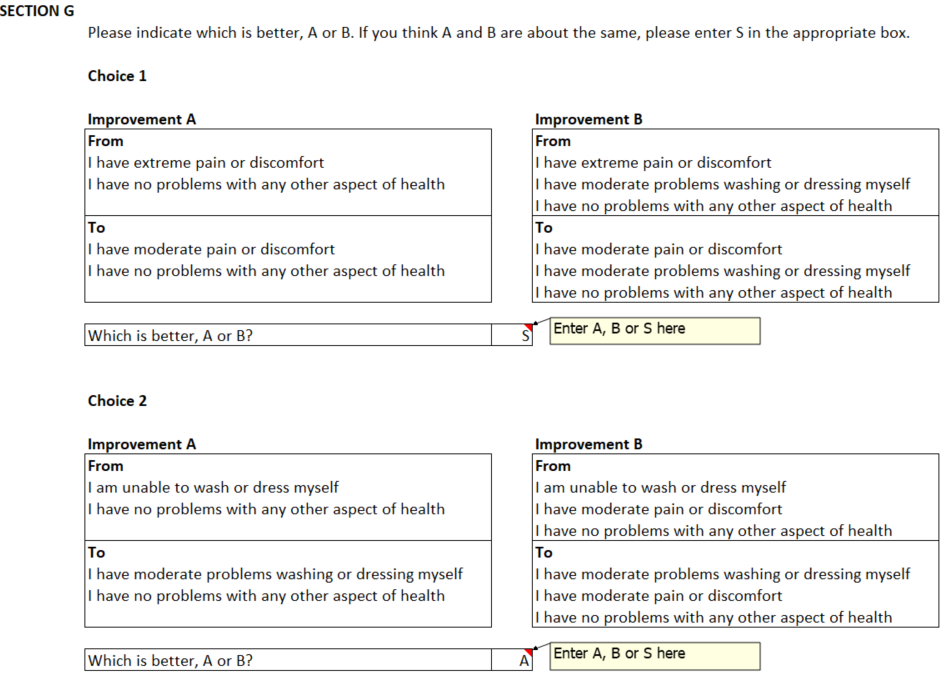

Supplement: Supplementary file 1 — Supplementary material 1 (DOCX 935 KB) [file 10198_2018_993_MOESM1_ESM.docx]
